# The Role of Alvarado Score in Predicting Acute Appendicitis and Its Severity in Correlation to Histopathology: A Retrospective Study in a Qatar Population

**DOI:** 10.7759/cureus.26902

**Published:** 2022-07-15

**Authors:** Mohannad Al-Tarakji, Ahmad Zarour, Rajvir Singh, Mohamed Said Ghali

**Affiliations:** 1 Acute Care Surgery, Hamad Medical Corporation, Doha, QAT; 2 General Surgery, Ain Shams University, Cairo, EGY

**Keywords:** acute appendicitis, histopathology (hp), negative appendectomy rate, severity of disease, alvarado score

## Abstract

Background/objective

Acute appendicitis (AA) is one of the most common surgical emergencies that require a proper diagnosis to avoid a negative outcome in the case of missed or delayed diagnosis. Our study aims to assess the diagnostic power of the Alvarado score and the prediction of the severity of acute appendicitis in correlation to intraoperative findings and the final histopathology (HP) result.

Methods

This retrospective study was applied to 1,303 patients with clinically proven acute appendicitis (AA) and available HP results. We correlated Alvarado score to the gold standard HP and intraoperative findings. We selected the cutoff point of Alvarado at 5 and 7 as they were the most frequent cutoff value mentioned in the literature and based on the ROC curve in this study to assess sensitivity, specificity, positive predictive value (PPV), and negative predictive value (NPV).

Results

The mean age of the study cohort is 33.3 ± 9.5 years, with a male predominance (75.8%). The negative appendectomy (NA) rate was 4%. The operative complication rate was 1.2%, and we recorded one mortality case (0.1%). The diagnostic evidence of AA was in 95.9% of cases. Alvarado score ≥ 7 presented sensitivity and specificity of 66.4% and 69.8%, respectively, with PPV of 98.1% and NPV of 8.1%, with an accuracy of 66.5%. For Alvarado score ≥ 5, the sensitivity was 91.2%, specificity was 22.6%, PPV was 96.5%, NPV was 9.8%, and accuracy was 88.4%. In addition, we demonstrated statistical significance between Alvarado risk stratification with HP and intraoperative grades (p = 0.001 each).

Conclusion

The Alvarado scoring system alone is not enough to diagnose AA with unsatisfactory sensitivity and specificity. However, it is a good indicator of the severity of AA that we can depend on to prioritize those patients waiting for surgery.

## Introduction

Acute appendicitis (AA) is caused by inflammation of the vermiform appendix with many hypotheses justifying this disease’s etiology. One of the main etiologies is obstruction of the appendicular lumen, whether by stool, vegetable seeds, foreign body, or neoplasm, which increases luminal pressure of the appendix with the subsequent impaired vascular supply of the appendicular wall and hence inflammation and perforation [1]. Another hypothesis is going with racial and genetic distribution, while another study is referring to environmental and socioeconomic distribution [2,3]. Most emergency departments worldwide reported AA as the most common surgical emergency they faced in daily activities [4]. The risk of getting this disease during a person’s lifetime is about 6%-9%, affecting the young population between the second and third decades of their lives [5,6]. Medical presentations range from abdominal pain to a life-threatening disease with peritonitis and septic shock. Therefore, early diagnosis of AA is the main factor that leads to the best outcome in managing this common disease [1,7]. The management can also be tailored according to the presentation from nonoperative with antibiotics to exploration laparotomy in delayed and neglected cases. Hence, its early diagnosis carries a significant challenge, especially with different presentations regarding symptoms and pain tolerance, which differ according to sex and race with a diversity of differential diagnoses, especially in females [8]. There are various ways to diagnose AA depending on clinical assessment, laboratory, and radiological results. Many scientists and medical schools invented diagnostic criteria and clinical scores for diagnosing AA, such as the Alvarado score, modified Alvarado score, pediatric appendicitis score, appendicitis inflammatory response score, adult appendicitis score, and Raja Isteri Pengiran Anak Saleha Appendicitis score [9-14]. The Alvarado score is one of the earlier used scoring systems for the diagnosis of acute appendicitis that depends on eight components with a total score of 10. Score 1 or 2 was given to each factor according to its specific correlation with appendicitis diagnosis. The components migratory pain to the right iliac fossa, lower right quadrant rebound tenderness, temperature more than 37.3°C, nausea or vomiting, anorexia, and increased neutrophilic count > 75% with a shift to the left were given a score of 1 each. The rest of the components, tenderness of the right iliac fossa and increase in leukocytes above 10,000 per µL, were scored 2 for each one. Alvarado risk stratification has a low probability for scores 1-4, an intermediate probability for scores 5-6, and a high probability for scores 7-10 for AA. According to risk stratification, we decided to proceed with surgical intervention, further investigation, or exclusion of the diagnosis of AA for high, intermediate, and low stratification groups, respectively [9,15].

The radiological assessment of AA with ultrasound (US) or computed tomography (CT) scan was left for cases with a borderline Alvarado score or a patient with confusing clinical presentation according to gender and clinical suspicion [16,17]. Many centers all over the world consider CT scans as their main diagnostic tool to avoid false-positive cases and decrease the rate of negative appendectomies to less than 5% of operated cases but with the risk of radiation hazards and its remote lifelong complication and risks, as well as with its impact of delay of management and its potential cost [18-20].

We tried to assess the diagnostic power of the Alvarado score in the current era of advanced radiological scans and its easy availability with increasing emergency flow. Accordingly, we will know the validity of its application in our emergency and avoid the radiation hazards of routine CT scans. In addition, we will assess the rate of negative appendectomy (NA) in this study cohort.

## Materials and methods

This study is a retrospective analysis of prospectively collected data at Hamad General Hospital, Qatar’s largest tertiary care facility in Doha. The study was conducted using the database from January 1, 2018, to January 31, 2019, and received approval from the Medical Research Center Institutional Review Board (IRB) of Hamad Medical Corporation, Doha, Qatar (protocol number MRC-01-19-454). The study was applied to 1,303 patients with clinically proven AA based on physician judgment of clinical signs and symptoms and confirmed by radiological scans. Our inclusion criteria are as follows: (1) patients ≥14 years old; (2) patients who were clinically diagnosed, admitted with AA, and underwent appendectomy; and (3) available postoperative histopathology results.

We used our electronic medical record (EMR) database Cerner to retrieve study data. The collected data in this study demonstrate three sets of preoperative and postoperative data. The first set included demographics, history, and clinical characteristics (age, gender, nationality, presenting symptoms of abdominal pain and duration, fever, anorexia, nausea, vomiting, change of bowel habits, smoking or alcohol consumption, BMI, and comorbidities such as diabetes mellitus (DM), hypertension (HTN), coronary artery disease (CAD), arterial fibrillation (AF), and chronic kidney disease (CKD)); signs of tenderness and rebound tenderness; vitals data (systolic blood pressure (SBP), diastolic blood pressure (DBP), pulse rate, body temperature, and oxygen (O2) saturation); and intensive care unit (ICU) admission and length of ICU stay. The second set of data demonstrated laboratory results (white blood cells (WBCs), neutrophil count, lymphocyte count, platelets, hemoglobin level (Hb), international normalization ratio (INR), creatinine, blood urea nitrogen (BUN), pH, base excess, serum C-reactive protein (CRP), serum lactate, serum albumin, and serum glucose) and the radiological findings of CT scan. Finally, the third set of data included surgical procedure details (evaluation to surgery time (EST), date/type of surgery, operative grading of appendicitis, conversion to open surgery, intraoperative complications, postoperative drain insertion, date of drain removal, postoperative antibiotics (AB), duration of AB course, postoperative imaging if there, reoperation, cause of reoperation (collection and bleeding), readmission and cause of readmission (PE, collection, stump appendicitis, fever, and abdominal pain) and death) and histopathology (HP) grading. The results of the study are presented in Table 1.

**Table 1:** Alvarado score. Alvarado risk stratification: score 1-4, low probability of acute appendicitis; score 5-6, intermediate probability of acute appendicitis; score 7-10, high probability of acute appendicitis

Alvarado components	Score points
Symptoms	
Migratory pain to the right iliac fossa	1
Anorexia	1
Nausea or vomiting	1
Signs	
Tenderness of the right iliac fossa	2
Rebound tenderness	1
Temperature more than 37.3°C	1
Laboratory	
Leukocytes above 10,000 per µL	2
Neutrophilic count > 75%	1

The Alvarado score was calculated according to the available data retrospectively after the surgical management of AA and the final HP results. The patients were stratified accordingly into three groups: group I with less probability of having AA with a score from 1 to 4, group II with intermediate probability with a score from 5 to 6, and group III with a high probability with a score above 7 to 10 (Table 1) [9].

We used the American Association for the Surgery of Trauma (AAST) grading for AA [21] as the nearest grading description to our recorded findings. We classified the HP microscopic findings to grade 0 for normal or no evidence of AA, grade I for mild form of AA, grade II for gangrenous/perforated AA, and grade III for AA with incidental neoplastic finding. Regarding operative findings, it was described as grade 0 for appendix with a normal appearance, grade I for nonperforated AA, grade II for gangrenous/impending perforation AA, grade III for perforated AA with fluid collection, grade IV for mass forming AA, and grade V for finding mentioned before with generalized peritoneal contamination. It was considered that grades I and II are uncomplicated AA and that grades III, IV, and V were considered complicated AA. We correlated the Alvarado score to the gold standard HP findings and intraoperative findings to assess the relation of the Alvarado scoring system to the severity of the results mentioned above. We chose the cutoff point of the Alvarado score to be at 5 and 7 to assess the sensitivity, specificity, positive predictive value (PPV), and negative predictive value (NPP).

Statistical methods

Descriptive statistics in the form of mean and standard deviation (SD) for interval variables and frequency with percentages for categorical variables were calculated according to the Alvarado and HP gradings. Chi-square tests were applied to see the association between HP and the Alvarado gradings. One-way ANOVAs were performed to find the mean differences among HP and Alvarado gradings for all interval variables. ROC curve and the concordance (c) statistic were used to assess the ability of the Alvarado score in the diagnosis of AA disease at different cutoff values. A p-value less than 0.05 (typically ≤ 0.05) was considered statistically significant. The Statistical Package for Social Sciences (SPSS) version 28.0 (IBM Corp., Armonk, NY, USA) was used for data analysis.

## Results

A total of 1,303 patients who underwent appendectomy for the diagnosis of AA were enrolled in the study. The mean age of the study cohort is 33.3 ± 9.5 years. The male-to-female ratio was 3.1:1 with a male predominance (75.8%). Regarding nationality, most of the study cases were from Asia (80.9%), followed by Africa (18.1%) and other continents (1%). The mean time between the onset of acute appendicitis symptoms to the diagnosis was 1.8 ± 1.5 days and the time from diagnosis of AA to surgery was 26.6 ± 11 hours. The most frequent presentations of factors establishing the Alvarado score were tenderness of the right iliac fossa (99.3%), rebound tenderness (79.9%), and an increase in leukocytes to >10,000 per µL (76.9%). The mean Alvarado score for the whole cohort was 7.0 ± 1.7, and this number is affected by the risk stratification of the whole cohort, which has been divided into three groups: less probability (group I) with 121 patients, intermediate probability (group II) with 336 patients, and high probability (group III) with 846 patients. Regarding laboratory findings, as demonstrated in Table 2 and Table 3, we recorded the mean serum CRP concentration for the cohort of 49 ± 69 mg/L, the mean WBC count was 13.9 ± 4.3 × 10^3^/uL, 43.8% of patients had a WBC count between 10,000 and 15,000 × 10^3^/uL, and 33.2% of the patients had WBCs > 15,000 × 10^3^/uL. Laparoscopic appendectomy was the main surgical procedure, representing 89.9% of the cases, while open appendectomy represented 10.1%. The conversion rate from a laparoscopic approach to an open approach was 0.6%. Fifty-three (4.1%) patients required intraoperative drain insertion. The mean days for the perioperative antibiotic requirement was 6.0 ± 4.0 days. The intraoperative findings recorded during surgery were as follows: 1.5% for grade 0, 76.9% for grade I, 14% for grade II, 0.4% for grade III, and 7.2% for grade IV. Fifty (3.8%) patients required postoperative imaging due to postoperative-related or non-related clinical findings. Reoperation was encountered in three (0.2%) patients; one patient was operated on for drainage and washout of abdominal collection, and the other two patients were operated on to control postoperative bleeding. The readmission rate was 1.5% (20 patients). One patient was admitted with pulmonary embolism, 14 patients were diagnosed with abdominal collection, one patient was diagnosed with stump appendicitis, one patient had a fever, and three patients had significant abdominal pain without definite pathology. The operative complication rate was 1.2%, the mean length of hospital stay was two days, and we recorded one mortality case (0.1%). Regarding HP findings, the diagnostic evidence of AA was in 95.9% of cases. There were 52 patients with a normal appendix, with no evidence of inflammation divided according to gender with 25 females and 27 males. Hence, the negative appendicectomy rate was 4%. CT scan was done for 1,095 (84%) patients, ultrasound scan (US) was recorded in 217 patients (16.7%), and 136 (10.4%) patients obtained both studies prior to surgery. The HP findings were grade 0 in 52 patients, grade I in 1,198, grade II in 41, and grade III for AA resulting from neoplastic findings in 11 cases. All details are demonstrated in Table 2 and Table 3.

**Table 2:** Demographics, clinical vitals, and laboratory results in relation to Alvarado risk stratification and histopathology grades. SD: standard deviation; IQR: interquartile range; BMI: body mass index; SBP: systolic blood pressure; DBP: diastolic blood pressure; WBC: white blood cells; Hb: hemoglobin; BUN: blood urea nitrogen; PH: potential of hydrogen; CRP: C-reactive protein, CT: computed tomography

	Alvarado			HP					p-value	
Variable (mean ± SD)	Group I (n = 121)	Group II (n = 336)	Group III (n = 846)	Grade 0 (n = 52)	Grade I (n = 1,198)	Grade II (n = 42)	Grade III (n = 11)	Total (n = 1,303)	Alvarado	HP
Age (years)	34 ± 10	32 ± 8.8	32 ± 10	31.4 ± 9.9	32.3 ± 9.5	34.3 ± 10.6	33.5 ± 9.6	33.3 ± 9.5	0.12	0.50
Hospital stay (days) (IQR)	2 (1-2)	1.5 (1-2)	2 (1-2)	2 (1-2)	2 (1-2)	3 (2-5)	2 (1-2)	2 (1-2)	0.25	0.001
Surgery waiting time (hours)	25.6 ± 12	25.8 ± 13	24 ± 11	26.7 ± 14.8	24.5 ± 11	22.5 ± 11.3	29.3 ± 4.6	26.6 ± 11	0.001	0.15
Duration of symptoms (days)	2.6 ± 2.7	1.9 ± 1.5	1.7 ± 1.2	3.4 ± 3	1.7 ± 1.4	1.9 ± 1.1	2.4 ± 1.4	1.8 ± 1.5	0.001	0.001
BMI	26.3 ± 4.7	25.7 ± 4.7	25.2 ± 4.9	25.9 ± 5	25.4 ± 4.8	26.7 ± 6	27 ± 8	25 ± 5	0.02	0.16
SBP (mmHg)	122 ± 14	119 ± 12	120 ± 13	118 ± 14	120 ± 13	120 ± 16	115 ± 9	120 ± 13	0.15	0.60
DBP (mmHg)	74.4 ± 8.9	73 ± 10	72 ± 9	20.9 ± 8.8	72.6 ± 9.5	73 ± 10	71 ± 5	73 ± 9	0.08	0.59
Pulse rate (beats/minutes)	77 ± 13	78 ± 12.9	81 ± 13	79.9 ± 13.5	79.5 ± 13	86.7 ± 14.9	77.6 ± 11	80 ± 13	0.003	0.006
Temperature (°Celsius)	36.8 ± 0.4	36.8 ± 0.5	36.9 ± 0.6	36.8 ± 0.6	36.9 ± 0.5	37 ± 0.6	36.7 ± 0.6	36.9 ± 0.6	0.001	0.001
Oxygen saturation	99 ± 0.9	99 ± 0.8	99 ± 1.2	99 ± 0.8	99 ± 1	98 ± 2.9	99 ± 0.8	99 ± 1.1	0.57	0.002
WBC (10^3^/uL)	8.2 ± 2.5	11.3 ± 4.3	15 ± 3.6	11.1 ± 4.5	13.4 ± 4.3	14.7 ± 5.3	12.4 ± 5	13.9 ± 4.3	0.001	0.001
Neutrophil count (10^3^/uL)	5.3 ± 1.9	8.3 ± 4.1	12.4 ± 3.5	7.7 ± 4.5	10.7 ± 4.2	12.4 ± 5	9.2 ± 4.7	10.6 ± 4.3	0.001	0.001
Lymphocyte count (10^3^/uL)	2.1 ± 0.9	2.1 ± 0.9	1.6 ± 0.9	2.3 ± 0.9	1.8 ± 1	1.3 ± 0.8	2.2 ± 1.4	1.8 ± 1	0.001	0.001
Platelet count (10^3^/uL)	245 ± 58	251 ± 63	259 ± 64	273 ± 59	255 ± 62	236 ± 79	293 ± 82	255 ± 63	0.02	0.007
Hb (gm/dL)	14 ± 1.7	14.3 ± 1.9	14.4 ± 1.7	13.5 ± 1.9	14 ± 1.7	14.2 ± 2.3	14 ± 2.2	14.3 ± 1.7	0.02	0.003
International normalized ratio (INR)	1 ± 0.1	1.2 ± 0.1	1.1 ± 0.2	1.1 ± 0.2	1.1 ± 0.1	1.3 ± 0.3	0.9 ± 0.08	1.1 ± 0.2	0.09	0.001
Serum creatinine (umol/L)	73.7 ± 16.4	72.5 ± 17.6	73.8 ± 23.9	66.5 ± 19.2	74 ± 22	79 ± 21	64 ± 14	73.5 ± 21.8	0.64	0.02
Serum BUN (umol/L)	3.8 ± 1.2	3.9 ± 2.6	4 ± 2.7	3.9 ± 1.4	3.9 ± 2.4	4.9 ± 6.1	3.3 ± 0.4	3.9 ± 2.6	0.82	0.05
pH	7.8 ± 0.03	7.4 ± 0.04	7.4 ± 0.04	7.4 ± 0.03	7.6 ± 0.04	7.4 ± 0.05	7.4 ± 0.03	7.4 ± 0.04	0.008	0.50
Base excess (mmol/L)	1.2 ± 1.6	0.9 ± 1.5	1 ± 1.5	1.1 ± 1.5	0.99 ± 1.5	0.7 ± 2.2	1 ± 1.9	1 ± 1.5	0.41	0.77
Serum CRP (mg/L)	31 ± 33	42 ± 61	53 ± 75	34.3 ± 55.9	46.1 ± 67	130 ± 98	14 ± 15.6	49 ± 70	0.03	0.001
Serum lactate	1.6 ± 0.6	1.8 ± 0.8	2.1 ± 0.9	1.5 ± 0.7	2 ± 0.9	2.3 ± 0.9	1.8 ± 1.1	1.9 ± 0.9	0.001	0.002
Serum albumin (gm/L)	39 ± 3.7	38.9 ± 4.4	39.5 ± 4.2	38 ± 3.9	39 ± 4.0	37 ± 4.5	37 ± 3.9	39 ± 4.2	0.34	0.001
Serum glucose (mmol/L)	5.9 ± 2.1	5.8 ± 1.3	6.4 ± 2.1	5.7 ± 1.2	6.2 ± 2.0	6.8 ± 1.9	5.8 ± 0.8	6.2 ± 1.9	0.001	0.06
Appendicular diameter (CT finding)	10.7 ± 2.4	10.5 ± 2.6	10 ± 2.8	9.1 ± 1.9	8.7 ± 1.8	11 ± 2.6	13.6 ± 3.0	10 ± 2.8	0.001	0.001
Alvarado score				5.8 ± 1.7	7.1 ± 1.7	7.6 ± 1.5	6.6 ± 2.2	7 ± 1.7		0.001

**Table 3:** Demographics and radiological and clinical characteristics in relation to Alvarado risk stratification and histopathology grades. HP: histopathology; DM: diabetes mellitus; HTN: hypertension; CAD: coronary artery disease, CKD: chronic kidney disease; AF: atrial fibrillation; WBC: white blood cells

	Alvarado			HP					p-value	
Variable (N (%))	Group I (n = 121)	Group II (n = 336)	Group III (n = 846)	Grade 0 (n = 52)	Grade I (n = 1,198)	Grade II (n = 42)	Grade III (n = 11)	Total (n = 1,303)	Alvarado	HP
Gender (male)	89 (73.6)	250 (74.4)	649 (76.7)	27 (51.9)	919 (76.8)	35 (83.3)	7 (63.6)	988 (75.8)	0.58	0.001
Nationality									0.96	0.02
Asian	97 (80.2)	268 (79.8)	689 (81.4)	47 (90.4)	968 (80.9)	34 (81)	5 (45.5)	1,054 (80.9)		
African	23 (19)	64 (19)	149 (17.6)	4 (7.7)	218 (18.2)	7 (16.7)	6 (54.5)	236 (18.1)		
Others	1 (0.8)	4 (1.2)	8 (0.9)	1 (1.9)	11 (0.9)	1 (2.4)	0 (0)	13 (1)		
Migratory abdominal pain	37 (30.6)	180 (53.6)	651 (77)	24 (46.2)	806 (67.3)	30 (71.4)	7 (63.6)	868 (66.6)	0.001	0.01
Fever	16 (13.2)	45 (13.4)	154 (18.2)	11 (21.2)	192 (16)	10 (23.8)	1 (9.1)	215 (16.5)	0.08	0.38
Anorexia	27 (22.3)	152 (45.6)	583 (68.9)	29 (55.8)	698 (58.3)	28 (66.7)	6 (54.6)	762 (58.5)	0.001	0.71
Nausea	37 (30.6)	179 (53.3)	670 (79.2)	35 (67.3)	810 (67.7)	31 (73.8)	9 (81.8)	886 (68)	0.001	064
Vomiting	25 (20.7)	147 (43.8)	581 (68.7)	29 (55.8)	687 (57.4)	31 (73.8)	5 (45.5)	753 (57.8)	0.001	0.15
Change in bowel habits	7 (5.8)	26 (7.7)	70 (8.3)	9 (17.3)	87 (7.3)	5 (11.9)	1 (9.1)	103 (7.9)	0.63	0.05
Smoking	10 (8.3)	29 (8.6)	70 (8.3)	1 (1.9)	101 (8.4)	6 (14.3)	1 (9.1)	109 (8.5)	0.98	0.19
Alcohol consumption	1 (0.8)	2 (0.6)	13 (1.5)	0 (0)	15 (1.3)	1 (2.4)	0 (0)	16 (1.2)	0.28	0.74
DM	7 (5.8)	15 (4.5)	42 (5)	4 (7.7)	58 (4.8)	1 (2.4)	1 (9.1)	64 (4.9)	0.84	0.60
HTN	10 (8.3)	7 (2.1)	44 (5.2)	3 (5.8)	51 (4.3)	5 (11.9)	2 (18.2)	61 (4.7)	0.10	0.02
CAD	1 (0.8)	0 (0)	3 (0.4)	1 (1.9)	2 (0.2)	1 (2.4)	0 (0)	4 (0.3)	0.34	0.01
CKD	1 (0.8)	0 (0)	3 (0.4)	0 (0)	3 (0.3)	1 (2.4)	0 (0)	4 (0.3)	0.34	0.10
AF	0 (0)	0 (0)	1 (0.1)	0 (0)	1 (0.1)	0 (0)	0 (0)	1 (0.1)	0.76	0.99
Blood culture	0 (0)	6 (1.8)	13 (1.5)	1 (1.9)	13 (1.1)	5 (11.9)	0 (0)	19 (1.5)	0.35	0.001
Surgery approach (laparoscopy)	115 (95)	313 (93.2)	743 (87.8)	52 (100)	1,072 (89.6)	37 (88.1)	9 (81.8)	1,170 (89.9)	0.003	0.08
Conversion	0 (0)	3 (0.9)	5 (0.6)	1 (1.9)	7 (0.6)	0 (0)	0 (0)	8 (0.6)	0.55	0.61
Operative complication	1 (0.8)	5 (1.5)	10 (1.2)	0 (0)	14 (1.2)	2 (4.8)	0 (0)	16 (1.2)	0.83	0.16
Postoperative drain	0 (0)	15 (4.5)	38 (4.5)	0 (0)	43 (3.6)	10 (23.8)	0 (0)	53 (4.1)	0.60	0.001
Postoperative imaging	3 (2.5)	10 (3)	37 (4.4)	3 (5.8)	39 (3.3)	6 (14.3)	2 (18.2)	50 (3.8)	0.38	0.001
Readmission	0 (0)	3 (0.9)	17 (2)	1 (1.9)	18 (1.5)	1 (2.4)	0 (0)	20 (1.5)	0.13	0.93
Reoperation	0 (0)	0 (0)	3 (0.4)	0 (0)	3 (0.3)	0 (0)	0 (0)	3 (0.2)	0.44	0.97
Mortality	0 (0)	0 (0)	1 (0.1)	0 (0)	1 (0.1)	0 (0)	0 (0)	1 (0.1)	0.76	0.99
WBCs (10,000-15,000 10^3^/uL)	75 (62)	186 (55.4)	309 (36.6)	42 (80.8)	528 (92.6)	0 (0)	0 (0)	570 (43.5)	0.001	0.001
WBCs (>15,000 10^3^/uL)	28 (23.1)	80 (23.8)	324 (38.3)	2 (3.8)	398 (33.2)	32 (76.2)	0 (0)	432 (33.2)	0.001	0.001

**Figure 1:**
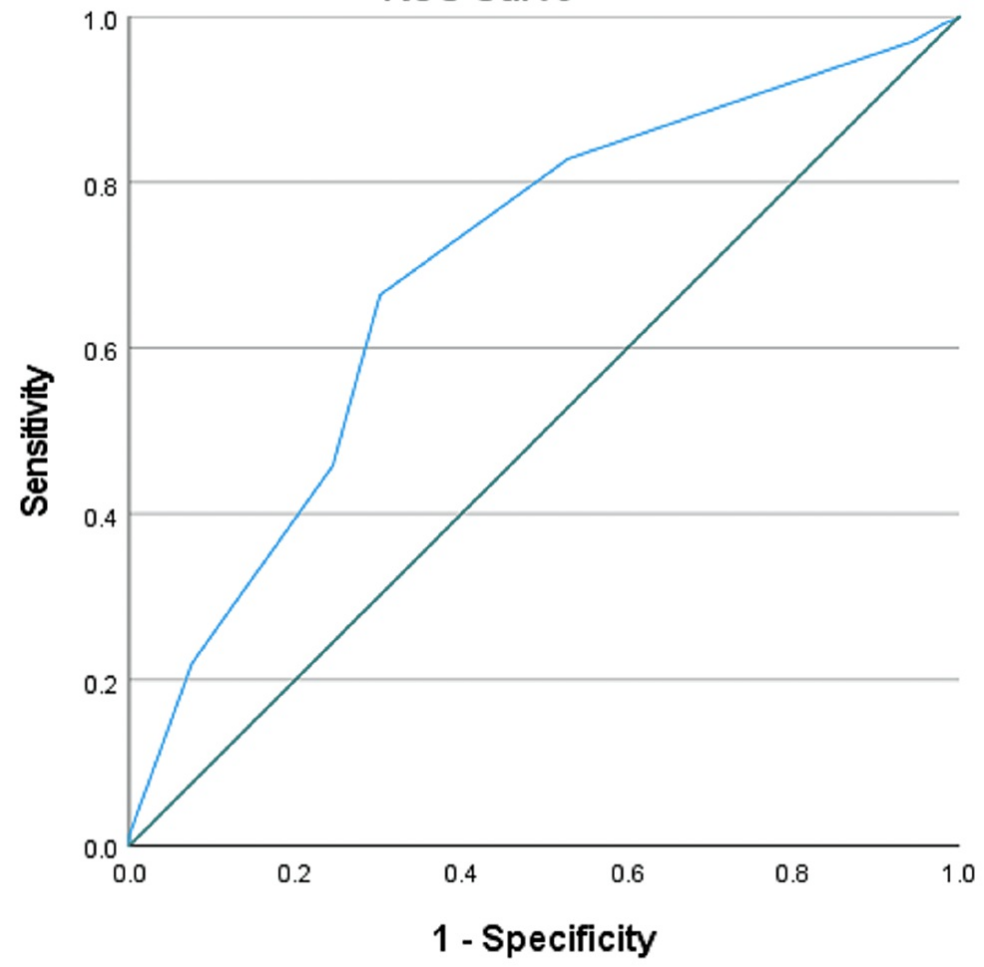
ROC curve. Diagonal segments are produced by ties.

For the evaluation of the Alvarado score, computing the sensitivity, specificity, positive predictive value (PPV), and negative predictive value (NPV) with the optimal cutoff value for the Alvarado score, we found a statistical significance between the Alvarado score and the diagnostic confirmation using a cutoff score greater than or equal to 5 and 7 (p = 0.003 and p≥0.001, respectively), showing a greater chance of AA diagnosis for such results; this was according to the ROC curve with an area under the curve of 0.696, which showed a cutoff greater than 5.5 as being the most significant (Figure 1).

Scores equal to or higher than 7 presented sensitivity and specificity of 66.4% and 69.8%, respectively, with a PPV of 98.1%, an NPV of 8.1%, and an accuracy of 66.5%. For scores greater than or equal to 5, the sensitivity was 91.2%, specificity was 22.6%, PPV was 96.5%, NPV was 9.8%, and accuracy was 88.4%.

Correlation between the Alvarado score stratification and the study variables

Of the 1,303 patients, 121 (9.3%) patients belonged to group I with a low probability Alvarado score of AA, which includes 89 (73.6%) males; 336 (25.8%) patients were under group II with 250 (76.7%) males; and 846 (64.9%) patients belonged to category III consisting of 649 (76.7%) males, which meant that, according to the Alvarado score, the disease is more common in male and presented more with group III. However, this observation may not reflect the truth as Qatar’s demography has high male proportions (75%) due to the influx of mainly male laborers [22]. There is statistical significance in vital data between Alvarado groups toward pulse rate and body temperature (p = 0.003 and p = 0.001, respectively). For the Alvarado score components, we found a statistical significance among the groups toward migratory abdominal pain, anorexia, nausea, and vomiting (p = 0.001 each). Regarding laboratory results, WBC count showed significant variability between the three groups (p = 0.001), with a higher level in high probability group III, the same with neutrophil count (p = 0.001). With lymphocyte count, a higher level is toward group I in comparison with group II and group III (p = 0.001). There was a significant finding for WBC brackets; WBCs > 15,000 × 10^3^/uL demonstrate more percentage with group III (p = 0.001), but as regards WBC count between 10,000 and 15,000, we found a higher rate with group I, followed by group II, and then group III (p = 0.001). Serum C-reactive protein (CRP), lactate, and glucose levels showed statistical significance with an increase in the probability of AA (p = 0.03, p = 0.001, and p = 0.001, respectively). According to CT scan findings, the appendicular diameter of the inflamed appendix demonstrated significance in group III more than in the other groups (p = 0.001). Regarding the surgical procedure offered, we found a significance between Alvarado risk groups, in which a more open appendectomy approach was used in the high-risk group III than in groups I and II (p = 0.003). The rest of the variables were nonsignificant (Table 2 and Table 3).

Correlation between the histopathological findings and the study variables

Out of the 1,303 patients in this study, grade 0 HP findings were found in 52 patients consisting of 51.9% male, grade I in 1,198 patients including 76.8% male, grade II in 41 cases with 83.3% male, and grade III in 11 cases with 63.6% male, demonstrating more male gender with the more severe form of appendicitis, which is statistically significant (p = 0.001). As mentioned earlier, this observation may not be accurate due to the predominance of the male gender in Qatar. In correlation with vital signs, pulse rates, raised temperature, and oxygen saturation in the blood have significant correlations (p = 0.006, p = 0.001, and p = 0.002, respectively). The duration of symptoms was more with the less severe noncomplicated HP finding (p = 0.001). Requirements for postoperative antibiotics and imaging, drain, and length of hospital stay (LOS) were significant with the complicated AA grade II more than other grades (p = 0.001 each).

The laboratory findings showed raising in WBCs and neutrophil count, which were significantly associated with high-grade HP of appendicitis than lower grades of inflammation (p = 0.001 each). Conversely, lymphocyte counts were lower in high grades than in lower grades (p = 0.001). The presence of an elevated CRP, INR, and lactate level was significantly associated with HP grade severity of appendicitis (p = 0.001, p = 0.001, and p = 0.002, respectively). Regarding Alvarado score components, migratory abdominal pain, raised body temperature, and abnormal WBCs were significant components between HP grades with a lower percentage in grade 0 representing no evidence of AA. Concerning CT scan findings, appendicular lumen diameter demonstrated statistical significance with HP disease severity (p = 0.001). The mean Alvarado score for patients with appendicitis was 7.1 ± 1.7, 7.6 ±1.5, and 6.6 ± 2.2 for grades I, II, and III of HP, respectively, while it was 5.8 ± 1.7 for those with normal histological findings (grade 0) (p = 0.001); this indicates that the higher the Alvarado score, the more severity the AA on histopathology examination. With regard to the association of HP with different components of the Alvarado score, migratory right iliac fossa pain (p = 0.01), abnormal WBC count (p = 0.001), and raised temperature (p = 0.001) were significantly correlated with HP grades. The rest of the compared variables were neither statistically nor clinically significant with HP grades (Table 2 and Table 3).

Correlation of the HP findings/operative findings with Alvarado risk stratification groups

We demonstrated statistical significance between Alvarado risk groups and HP grades (p = 0.001). It showed that about 52 patients with negative evidence of AA were distributed according to the Alvarado groups, more in group I than in groups II and III (9.9%, 7.4%, and 1.8%, respectively). This shows that Alvarado risk stratification is highly significant with HP finding (gold standard) in diagnosing acute appendicitis. This means a higher Alvarado score is related to lower negative appendectomy rates. Also, the higher the Alvarado score, the higher the HP grades and the more severe the inflammation is (Table 4). Regarding operative findings, we demonstrated a significant relationship between it and Alvarado risk groups (p = 0.001); a more severe operative finding is associated with the high Alvarado score risk group (Table 5).

**Table 4:** Histopathology (HP) findings in association with Alvarado groups.

Histopathology (HP)	Alvarado				p-value
N (%)	Group I (n = 121)	Group II (n = 336)	Group III (n = 846)	Total	0.001
Grade 0	12 (9.9)	25 (7.4)	15 (1.8)	52 (4)	
Grade I	108 (88.4)	299 (89)	791 (93.6)	1,198 (92)	
Grade II	1 (0.8)	8 (2.4)	33 (3.9)	42 (3.2)	
Grade III	1 (0.8)	4 (1.2)	6 (7.1)	11 (0.8)	
Total	122 (100)	336 (100)	845 (100)	1,303 (100)	

**Table 5:** Operating room (OR) findings in relation to Alvarado groups.

OR grades	Alvarado				p-value
	Group I (n = 121)	Group II (n = 336)	Group III (n = 846)	Total	0.001
OR grade 0	3 (2.5)	11 (3.3)	5 (0.6)	19 (1.5)	
OR grade I	100 (82.6)	274 (81.6)	628 (74.2)	1,002 (77)	
OR grade II	7 (5.8)	27 (8.0)	149 (17.6)	183 (14)	
OR grade III	0 (0)	1 (0.3)	4 (0.5)	5 (0.4)	
OR grade IV	11 (9.1)	23 (6.8)	60 (7.1)	94 (7.1)	
Total	121 (100)	336 (100)	846 (100)	1,303 (100)	

## Discussion

AA is one of the most common surgical diseases in the emergency department, and most of the time, it mimics and simulates other intra-abdominal surgical and medical pathologies. As it is an acute disease that carries a risk of complications and may be life-threatening if left without a prompt diagnosis, surgeons offer a surgical option rather than observation when the diagnosis of AA is suspicious to avoid such risks [7,8]. Negative appendicectomies carry additional challenges and burdens to the surgeons, patients, and healthcare facilities with non-indicated procedures. An accurate diagnosis of AA is required to avoid such conflicts. Therefore, many diagnostic scores were raised to help in diagnosis and to decrease negative appendectomy rates [20]. Alvarado score was one of the earliest score systems introduced to diagnose AA, and it managed to decrease non-indicated CT scans that carry radiation hazards in addition to its cost and availability.

This study included 1,303 patients operated on with a diagnosis of AA with available postoperative HP reports. We retrospectively studied the available data aiming to validate Alvarado score in correlation to gold standard HP findings. Across our sample, AA was found more in males (75.8%) than in females in Qatar’s population, which is similar to many reports in the literature [23,24]. On the other hand, other studies showed equal distribution between males and females [25,26]. In the view that AA affects the young population between the second and fourth decades, we found that our study cohort matches this with a mean age of 33.3 years, while a recently published article showed a relatively younger age group than this study that showed a mean age less than 30 years [17,27].

The negative appendectomy (NA) rate on HP results was 4% in this study, which is considered favorable in comparison to the literature rate [28-31]. In addition, we found that the female and male gender (51.9% and 58.1%, respectively) are equally in obtaining NA in this study, which is entirely different from other studies that confirmed that the NA rate was more prevalent in the female gender [32-34]. The most frequent Alvarado score components were tenderness of the right iliac fossa (99.3%), followed by rebound tenderness and leukocytosis, which are comparable to other studies [35,36]. However, it showed differences between the study of Swami et al. [37], who reported a lower incidence of leukocytosis, and Rodrigues et al. [38], where an elevated temperature was the most predominant. In our view, those differences in frequency are related to the patient’s status at the time of examinations and the differences in the cohort population and gender.

Most of the study cohort scored above Alvarado score of 5 (91%), and about 65% of the study population obtained a score of 7. Other studies showed a similar finding that most of the study population presented with a score above 5 [33,36,39,40]. We examined the sensitivity and specificity of the Alvarado score at the cutoff point of 5 and 7. We found that sensitivity, specificity, PPV, and NPV were 91.20%, 22.64%, 96.5%, and 9.8%, respectively, at the cutoff of 5, and sensitivity, specificity, PPV, and NPV were 66.40%, 69.81%, 98.1%, and 8.1%, respectively, at a cutoff point of 7. Recent studies demonstrated roughly similar results. Pifeleti et al. [27] reported that sensitivity, specificity, PPV, and NPV at Alvarado score ≥ 5 were 91.97%, 50%, 98.44%, and 15.38%, respectively, and at Alvarado score ≥ 7 were 63.5%, 75%, 98.86%, and 5.66%, respectively. Also, do Nascimento et al. [36] demonstrated a sensitivity of 88.17%, specificity of 37.5%, PPV of 94.25%, and NPV of 21.43% for Alvarado score ≥ 5, and a sensitivity of 38.71%, specificity of 87.5%, PPV of 97.3%, and NPV of 10.94% for Alvarado score ≥ 7. It was noticed that with the increase in the cutoff point, the sensitivity decreased, but the specificity increased. Another study displayed Alvarado score validation at cutoff value ≥ 7 and stated lower sensitivity of Alvarado score of 54%, specificity of 75%, PPV of 90%, and NPP of 29%, which concluded that the Alvarado score was not a helpful method in the diagnosis of AA [41].

We find in this study a significant correlation between HP and Alvarado risk stratification groups (p = 0.001) (Table 4). The highest percentage of different grades of HP were found mainly in group III of Alvarado; also, we noticed that false-positive appendicitis (negative appendectomy on HP) is higher in the lower Alvarado groups than in the higher groups, which is similar to other studies [36,42-44]. In terms of operative findings, we noticed a significant correlation with Alvarado, more percentage of AA along with the increase in Alvarado risk groups, especially with advanced grades of operative findings. Other studies also noticed this, which confirms the association between operative findings and Alvarado [35]. Other studies did not find such a correlation with Alvarado (Table 5) [45].

The mean time of evolution until the patients underwent appendectomy was 26.6 ± 11 hours, which is better than the study done by Sousa-Rodrigues et al., which reported a mean evaluation time of 32.4 ± 5.4 hours [45]. In this study, there is no statistical significance between Alvarado groups and the mean time of evaluation (p = 0.15), a similar finding found in the literature [36,46], but other studies showed a significant relationship between increased complication rate and longer evaluation time [42]. Our rate of complications was 1.2%, which is acceptable, and those complications mainly happened in relation to suppurative appendicitis in final HP, as displayed by other studies [36,47].

This study has limitations. Retrospective medical record reviews have inherent limitations in terms of data quality. Most of the study cohort obtained radiological assessment during emergency evaluation, which affected the accuracy of the negative appendectomy rate. The operating surgeons are different in degree and experience, which may affect recorded intraoperative findings. Future research could benefit from exploring such limitations. Nevertheless, the study has many strengths. A larger sample size gives a more accurate relation between variables. To our knowledge, it is the first to appraise the correlation between the Alvarado score and HP findings in Qatar, employing a wide range of interlacing variables in such a large sample.

## Conclusions

Our study results showed unsatisfactory sensitivity and specificity of the Alvarado score, and we conclude that the Alvarado score is not the sole valid tool to be used alone in diagnosing AA with many differential diagnoses, especially in females. However, the Alvarado score showed to be a good severity indicator of appendicitis to depend on while prioritizing patients waiting for surgery.
